# FGF21 attenuates neuroinflammation following subarachnoid hemorrhage through promoting mitophagy and inhibiting the cGAS-STING pathway

**DOI:** 10.1186/s12967-024-05239-y

**Published:** 2024-05-08

**Authors:** Yue Ma, Zhiqin Liu, Lele Deng, Jingjing Du, Zenghui Fan, Tian Ma, Jing Xiong, Xue Xiuyun, Naibing Gu, Zhengli Di, Yu Zhang

**Affiliations:** 1 Department of Neurology, The affiliated Xi’an Central Hospital of Xi’an Jiaotong University College of Medicine, Xi’an, 710032 Shaanxi China; 2 Department of Scientific Research Section, Tangdu Hospital, Air Force Medical University, Xi’an, 710038 Shaanxi China; 3 Department of Neurosurgery, Tangdu Hospital, Air Force Medical University, Xi’an, 710038 Shaanxi China

**Keywords:** Subarachnoid haemorrhage, FGF21, Microglia, mtDNA, cGAS-STING, Mitophagy

## Abstract

**Background:**

Subarachnoid hemorrhage (SAH) represents a form of cerebrovascular event characterized by a notable mortality and morbidity rate. Fibroblast growth factor 21 (FGF21), a versatile hormone predominantly synthesized by the hepatic tissue, has emerged as a promising neuroprotective agent. Nevertheless, the precise impacts and underlying mechanisms of FGF21 in the context of SAH remain enigmatic.

**Methods:**

To elucidate the role of FGF21 in inhibiting the microglial cGAS-STING pathway and providing protection against SAH-induced cerebral injury, a series of cellular and molecular techniques, including western blot analysis, real-time polymerase chain reaction, immunohistochemistry, RNA sequencing, and behavioral assays, were employed.

**Results:**

Administration of recombinant fibroblast growth factor 21 (rFGF21) effectively mitigated neural apoptosis, improved cerebral edema, and attenuated neurological impairments post-SAH. Transcriptomic analysis revealed that SAH triggered the upregulation of numerous genes linked to innate immunity, particularly those involved in the type I interferon (IFN-I) pathway and microglial function, which were notably suppressed upon adjunctive rFGF21 treatment. Mechanistically, rFGF21 intervention facilitated mitophagy in an AMP-activated protein kinase (AMPK)-dependent manner, thereby preventing mitochondrial DNA (mtDNA) release into the cytoplasm and dampening the activation of the DNA-sensing cyclic GMP-AMP synthase (cGAS)-stimulator of interferon genes (STING) signaling pathway. Conditional knockout of STING in microglia markedly ameliorated the inflammatory response and mitigated secondary brain injuries post-SAH.

**Conclusion:**

Our results present the initial evidence that FGF21 confers a protective effect against neuroinflammation-associated brain damage subsequent to SAH. Mechanistically, we have elucidated a novel pathway by which FGF21 exerts this neuroprotection through inhibition of the cGAS-STING signaling cascade.

## Introduction

Subarachnoid hemorrhage (SAH) is a critical medical emergency initiated by the rupture of aneurysms within the subarachnoid space [1, 2]. SAH gives rise to devastating neurological deficits and leads to mortality rates as high as 35% among patients [3, 4]. In recent years, it has been postulated that early brain injury (EBI) constitutes the primary contributor to the unfavorable prognosis associated with SAH [5–7]. Regrettably, no effective pharmacological intervention has been discerned to impede the progression of EBI. Neuroinflammation has been substantiated as a pivotal participant in the advancement of EBI subsequent to SAH [8–10]. It is widely acknowledged that microglia, the key innate immune cells of the brain, promptly undergo activation in response to diverse acute brain injuries [11, 12]. In a multitude of neurological disorders, attenuation of microglial inflammatory response has proven efficacious in diminishing acute brain injuries and ameliorating neurological outcomes [13, 14]. Hence, the identification of novel drug targets for modulating the pathogenic activities of microglia may constitute a viable approach to mitigate EBI.

Fibroblast growth factor 21 (FGF21), a widely recognized endocrine hormone, featured for its ability to enhance lipid mobilization and insulin sensitivity [15, 16]. A plethora of accumulating evidence to date has indicated that FGF21 exhibits protective effects on various disease, including diabetes, atherosclerosis, age, and central nerve system (CNS) disorders [17–20]. Unlike other members of the FGF family, FGF21 displays minimal mitogenic effects and possesses the unique capability to traverse the blood-brain barrier (BBB) owing to its low binding affinity with heparin [21, 22]. Notably, FGF21 can exerts its functions through interaction with FGFR1 that is widely expressed in brain cells, including microglia [23]. Consequently, FGF21 holds the potential to exert a profound impact on microglial activities. Despite the growing attention directed towards unraveling the interplay between FGF21 and brain function, the potential effects of FGF21 on SAH and the underlying molecular mechanisms remain enigmatic, as no comprehensive investigations have been conducted to date.

cGAS, the primary innate immune sensor for detecting double-stranded DNA (dsDNA), catalyzes the production of cyclic GMP–AMP, which in turn, activates STING, a crucial mediator of the interferon response. STING recruits TBK1 and facilitates its autophosphorylation, ultimately activating type I interferon (IFN-I) genes expression [24–26]. cGAS–STING pathway has been demonstrated to be involved in pathogenic process of various CNS disorders including Huntington’s disease, amyotrophic lateral sclerosis (ALS), Parkinson’s disease, as well as acute brain injuries like stroke [27–30]. Recent investigations have demonstrated that cytosolic mtDNA can trigger cGAS-STING signaling [31, 32]. However, it remains unclear concerning the potential role of cGAS-STING signaling in SAH. Therefore, a comprehensive exploration of the regulatory network and potential intervention strategies is warranted.

In this study, our objective was to determine the potential therapeutic effects of rFGF21 on EBI in a mouse model of SAH. Additionally, if such effects were observed, we aimed to elucidate the underlying molecular mechanisms accounting for the actions of rFGF21 in the context of SAH.

## Materials and methods

### Animals and ethics

Male C57BL/6J mice, aged 8 weeks and weighing between 20 and 25 g, were provided by the Animal Center of the Air Force Medical University. STING-floxp mice were procured from the esteemed Shanghai Model Organisms Center, Inc., CX3CR1-CreERT2 mice were obtained from the reputable Gempharmatech Co., Ltd. These mice were housed in a specific pathogen-free (SPF) environment, maintained at a temperature of 23 °C with 60% humidity, and subjected to a 12-hour light/dark cycle. Prior to commencing the subsequent experiments, the mice underwent a 2-week period of acclimation, during which they were provided with enough food and water. In the first section, experiments involving immunofluorescence staining, western blot, brain edema measurement, and qPCR assay each utilized 18 wild-type C57BL/6J mice (6 mice each from the Sham group, SAH group, and SAH + rFGF21 group). Neurobehavioral tests utilized 36 mice (12 mice each from the Sham group, SAH group, and SAH + rFGF21 group). In the second section, experiments encompassed immunofluorescence staining, western blot, brain edema measurement, and qPCR each utilized 30 mice (6 mice each from Sting^fl/fl^ + Sham group, Sting^fl/fl^ + SAH group, Sting^CKO^ + Sham group, Sting^CKO^ + SAH group, and Sting^CKO^ + SAH + rFGF21 group). Neurobehavioral tests involved 60 mice (12 mice each from Sting^fl/fl^ + Sham group, Sting^fl/fl^ + SAH group, Sting^CKO^ + Sham group, Sting^CKO^ + SAH group, and Sting^CKO^ + SAH + rFGF21 group). All experimental procedures adhered strictly to the guidelines outlined in the National Institutes of Health Guide for the Care and Use of Laboratory Animals, and were approved by the Animal Experiment Center of Air Force Military Medical University (IACUC-20,220,555).

.

### SAH surgery

SAH mouse model was established using the endovascular perforation technique, as described previously [33]. Briefly, following intraperitoneal administration of ketamine/xylazine for anesthesia, the carotid artery was meticulously exposed. A fine and sharp 6–0 monofilament nylon suture was skillfully employed to puncture the bifurcation point of the anterior and middle cerebral arteries. Subsequently, mouse was carefully placed in a heated chamber to facilitate post-operative recovery. As for the sham-treated mice, they underwent identical procedures, albeit without the actual perforation of the arterial vessel.

### Measurement of brain water content

Brain water content was assessed using the wet/dry method [34]. The brain tissues were removed and promptly weighed to determine its wet weight, subsequently subjected to desiccation in an oven maintained at 95 to 100 °C for a duration of 72 h. The brain tissues were then re-weighed to ascertain its dry weight. Brain water content = ([wet weight − dry weight]/wet weight) × 100%.

### Neurobehavioral tests

Neurological function was evaluated through the modified Garcia scale test [33]. This scoring system encompassed six measurements including spontaneous activity, spontaneous movement of four limbs, body proprioception, climbing, forepaw outstretching, and response to whisker stimulation, with a higher score indicating superior neurobehavioral outcomes. Neurobehavioral tests were performed in a blinded manner.

### Immunofluorescence staining

After a 72-hour post-SAH, mouse was sacrificed by an overdose of 2% pentobarbital sodium and underwent trans-cardiac perfusion with a 4% paraformaldehyde solution. Brain tissue was then removed and stored overnight in 4% paraformaldehyde. Dehydration was carried out using 30% sucrose solutions. Subsequently, brain sections measuring 15 μm were obtained and incubated in PBS solution containing 0.1% Triton X-100 for 30 min. To prevent non-specific binding, the sections were blocked with PBS solution containing 5% goat serum for an additional 30 min. Following this, the sections were incubated overnight at 4 °C with primary antibodies: IBA1 (ab289874, 1:300, Abcam) and CD68 (ab283654, 1:300, Abcam). Post-incubation, brain sections were thoroughly rinsed in PBS with three consecutive 5-minute washes. Subsequently, the sections were incubated at 25 °C for 1 h with corresponding secondary antibodies: Alexa Fluor 488 Anti-rabbit IgG (A11034, 1: 1000, Invitrogen) and Alexa Fluor 594 Anti-goat IgG (A11058, 1:1000, Invitrogen). Finally, the sections were stained with DAPI solution and observed using an A1 Si confocal microscope.

### TUNEL staining

Cell death was assessed by an unbiased observer utilizing the In Situ Cell Death Detection Kit (Roche, Germany) following the manufacturer’s guidelines. Following treatment with 0.3% hydrogen peroxide (0.5 h) and incubation with 0.25% proteinase K (0.5 h, 37℃), the sections were stained using the TUNEL reaction solution in a lightless environment (1 h, 37℃). Finally, brain sections were stained with DAPI solution and observed using an A1 Si confocal microscope.

### Quantitative real-time PCR

Total RNA was extracted using TRIzol Reagent (Invitrogen, Carlsbad, California, USA) in accordance with the manufacturer’s protocol. Subsequently, 1 mg of total RNA was reverse-transcribed using the HiScript II Q RT SuperMix for quantitative real-time PCR (qRT-PCR) (R223-01, Vazyme biotech). The qRT-PCR was performed using an iQTM 5 Optical Module Real-Time PCR Detection System (Bio-Rad, USA). ChamQTM SYBR qPCR master mix (Q311-02, Vazyme biotech) was utilized to quantify mRNA as per the manufacturer’s instructions. The mRNA levels were normalized to β-actin and calculated using the 2-ΔΔCt method.

### Isolation of microglia

Animal subjects were sacrificed by an overdose of 2% pentobarbital sodium and their brains were carefully extracted and dissected. To dissociate the tissues, a digestion process using Papain (2 mg.ml^− 1^, Worthington) in RPMI 1640 medium (Gibico) was carried out at 37℃ for 1 h. The resulting dispersed cells were then filtered using the 70 mm nylon mesh. Subsequently, the resulting cells were further resuspended with a 30% Percoll density gradient (GE Healthcare) and centrifuged at 900 g for 25 min. The cells were isolated by collecting the bottom fraction in the 30% Percoll solution. The samples were then treated with FcR Blocking Reagent (Miltenyi Biotec) for blocking and subjected to flow cytometry analysis for the detection of surface antigens. For fluorescence acquisition, the cells were stained with FITC anti-mouse CD11b Antibody (BioLegend) and PerCp-CyTM5.5 anti-mouse CD45 Antibody (BD Pharmingen). Flow cytometry (FACS Aria II SORP, BD Biosciences, USA) was employed for fluorescence acquisition, and the samples were gated based on CD11b + CD45dim expression, which represents the microglia population.

### Western blot analysis

Brain samples were homogenized using an ultrasonic homogenizer in a lysis buffer containing 1% protease inhibitor. After centrifugation of the lysate, the supernatant was collected and quantified using the BCA Protein Assay Kit (Thermo Scientific). Protein samples was then isolated by SDS-PAGE with 20 µg of protein loaded per well. Subsequently, protein was transferred onto PVDF membranes (IPVH-00010, Millipore Corporation, Billerica, MA, USA). The membranes were then incubated with 5% non-fat milk in TBST for 2 h. Primary antibodies, including anti-cGAS (NBP3-16666; Novus), anti-STING (D2P2F; Cell Signaling Technology), anti-phospho-STING (D8F4W; Cell Signaling Technology), anti-TBK1 (D1B4; Cell Signaling Technology), anti-phospho-TBK1 (D52C2; Cell Signaling Technology), anti-NF-κB (80979-1-RR; Proteintech), anti-p-NF-κB (93H1; Cell Signaling Technology), anti-IRF3 (11312-1-AP; Proteintech), anti-p-IRF3 (D6O1M; Cell Signaling Technology), anti-SQSTM1/p62 (D1Q5S; Cell Signaling Technology), anti-TIM50 (#62,317; Cell Signaling Technology), anti-Tom20 (D8T4N; Cell Signaling Technology) and anti-β-actin (13E5; Cell Signaling Technology), were then incubated with the membranes. Afterward, the membranes were washed three times with TBST for 5 min each and subsequently incubated with secondary antibodies. Lastly, protein bands were visualized using a Bio-Rad imaging system (Bio-Rad, USA).

### Statistical analysis

All statistical analyses were carried out using SPSS software (version 21.0). Statistical comparisons between the two groups were conducted using an unpaired, two-tailed Student’s t-test. For comparisons among multiple groups, a one-way or two-way analysis of variance (ANOVA) followed by the Student-Newman-Keuls test was employed. The rating scale data, specifically neurological scores, were analyzed using Kruskal-Wallis one-way ANOVA on ranks, followed by Steel-Dwass multiple comparisons. The results were expressed as the median with the 25th and 75th percentiles. The data were presented as means ± standard deviation (SD). A *p*-value of less than 0.05 was considered statistically significant.

## Results

### rFGF21 treatment alleviated brain edema, improved neurological function and reduced neural apoptosis following SAH

**Fig. 1:**
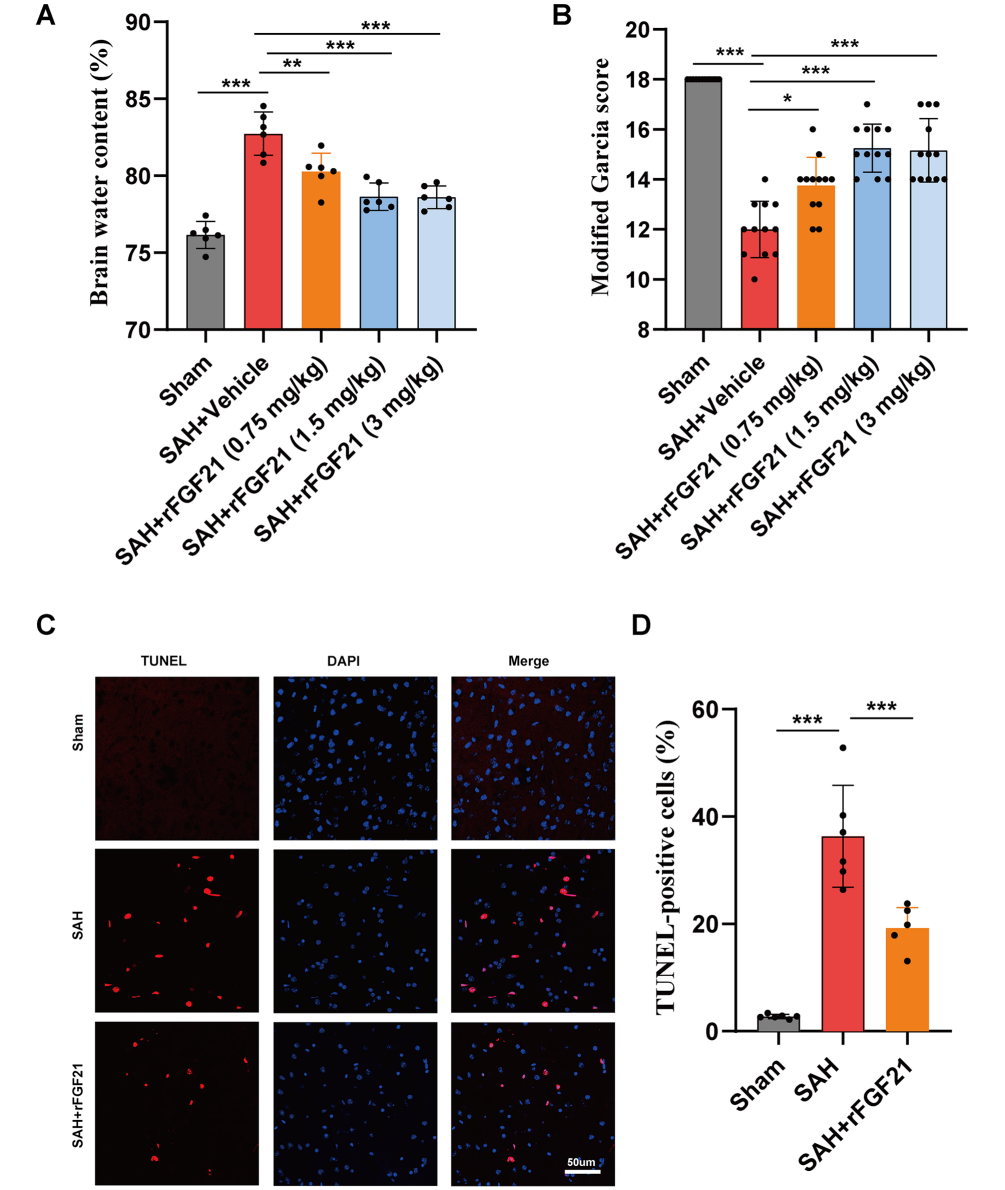
The effects of rFGF21 treatment on brain injuries after SAH. (**A**) Quantification of brain water content of mouse in each group (*n* = 6 per group). (**B**) Modified Garcia score in different experimental groups (*n* = 12 per group). (**C-D**) Representative TUNEL staining images and quantification of TUNEL-positive cells in mouse brain (*n* = 6 per group). Data are presented as means ± SD. *p < 0.05, **p < 0.01, ***p < 0.001

In our preliminary experimentation to determine the optimal FGF21 concentration, guided by prior research on the therapeutic application of FGF21 in CNS disorders [20, 35, 36], we administered three distinct doses (0.75 mg/kg, 1.5 mg/kg, and 3 mg/kg) of FGF21 to assess their efficacy in ameliorating cerebral edema and neurological impairment post-SAH. Evaluation of brain water content revealed that all three dosages of rFGF21 treatment effectively alleviated SAH-induced cerebral edema (Fig. 1A). Notably, the protective effect of 1.5 mg/kg was more pronounced than that of 0.75 mg/kg, with further dose escalation conferring no significant additional benefit. In line with these observations, rFGF21 treatment in a similar dosing regimen significantly mitigated SAH-induced neurological deficits (Fig. 1B). Consequently, 1.5 mg/kg rFGF21 was selected as the minimal effective dose for subsequent investigations. Moreover, TUNEL staining indicated a significantly increased number of apoptotic cells after SAH, which was markedly reduced upon additional administration of rFGF21 (Fig. 1C-D). Collectively, these results provide compelling evidence that rFGF21 mitigates apoptosis, cerebral edema, and neurological deficits following SAH.

### rFGF21 exhibited a mitigating effect on neuroinflammation subsequent to SAH

**Fig. 2:**
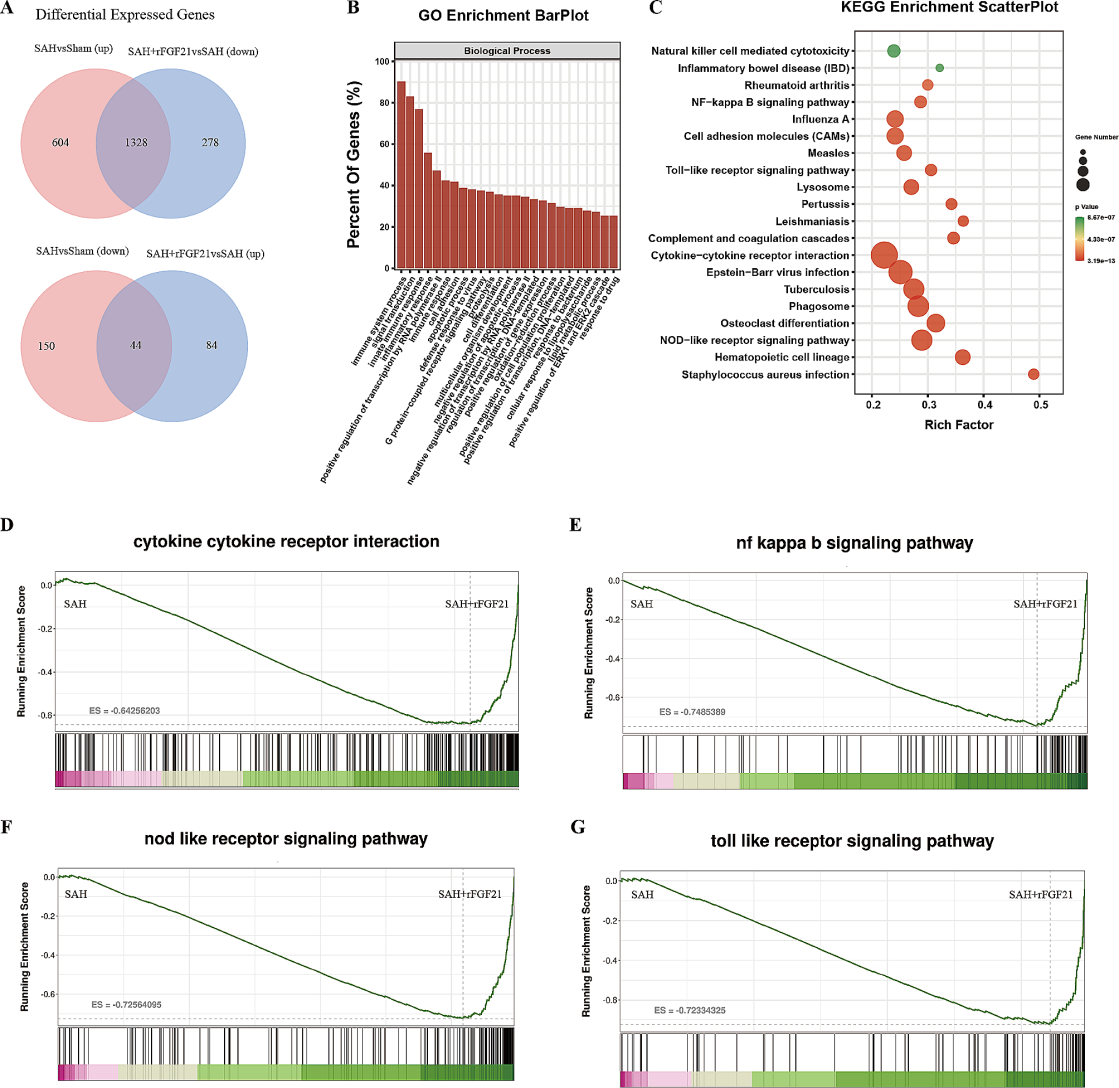
FGF21 attenuated neuroinflammation injury after SAH. (**A**) Venn diagram showed that 1372 shared genes overlapping between those differentially expressed genes were reversed by rFGF21 treatment. (**C**) Go enrichment analysis of the overlap of genes in the Venn diagram. (**C**) KEGG enrichment analysis of the overlap of genes in the Venn diagram. (**D-G**) GSEA plot of the NF − kappa B signaling pathway, Toll − like receptor signaling pathway, Cytokine − cytokine receptor interaction, and NOD-like receptor signaling pathway (SAH + rFGF21 versus SAH)

To delineate the molecular mechanisms underlying the neuroprotective attributes of FGF21, we performed bulk RNA-sequencing analysis on cerebral tissue obtained from Sham, SAH, and SAH + rFGF21 mice. Out of the 1932 genes identified, a total of 199 were downregulated and 1328 were upregulated in the cerebral tissue post-SAH. A Venn diagram depicted the reversal of 1328 upregulated genes and 44 downregulated genes induced by SAH following rFGF21 administration (Fig. 2A). Gene ontology (GO) analysis of these reversed genes induced by rFGF21 treatment unveiled pivotal biological processes enriched in immune response and inflammatory response (Fig. 2B). Moreover, KEGG analysis revealed notable changes in the expression of genes linked to inflammation-related pathways, including the NF − kappa B signaling pathway, Toll − like receptor signaling pathway, Cytokine − cytokine receptor interaction, and NOD-like receptor signaling pathway, upon rFGF21 treatment (Fig. 2C). Additionally, GSEA indicated the activation of these inflammation-related pathways following SAH, which were subsequently suppressed after supplemental rFGF21 treatment (Fig. 2D-G). Collectively, these findings indicate that rFGF21 significantly suppresses neuroinflammation after SAH.

### rFGF21 suppressed microglial activation following SAH

**Fig. 3:**
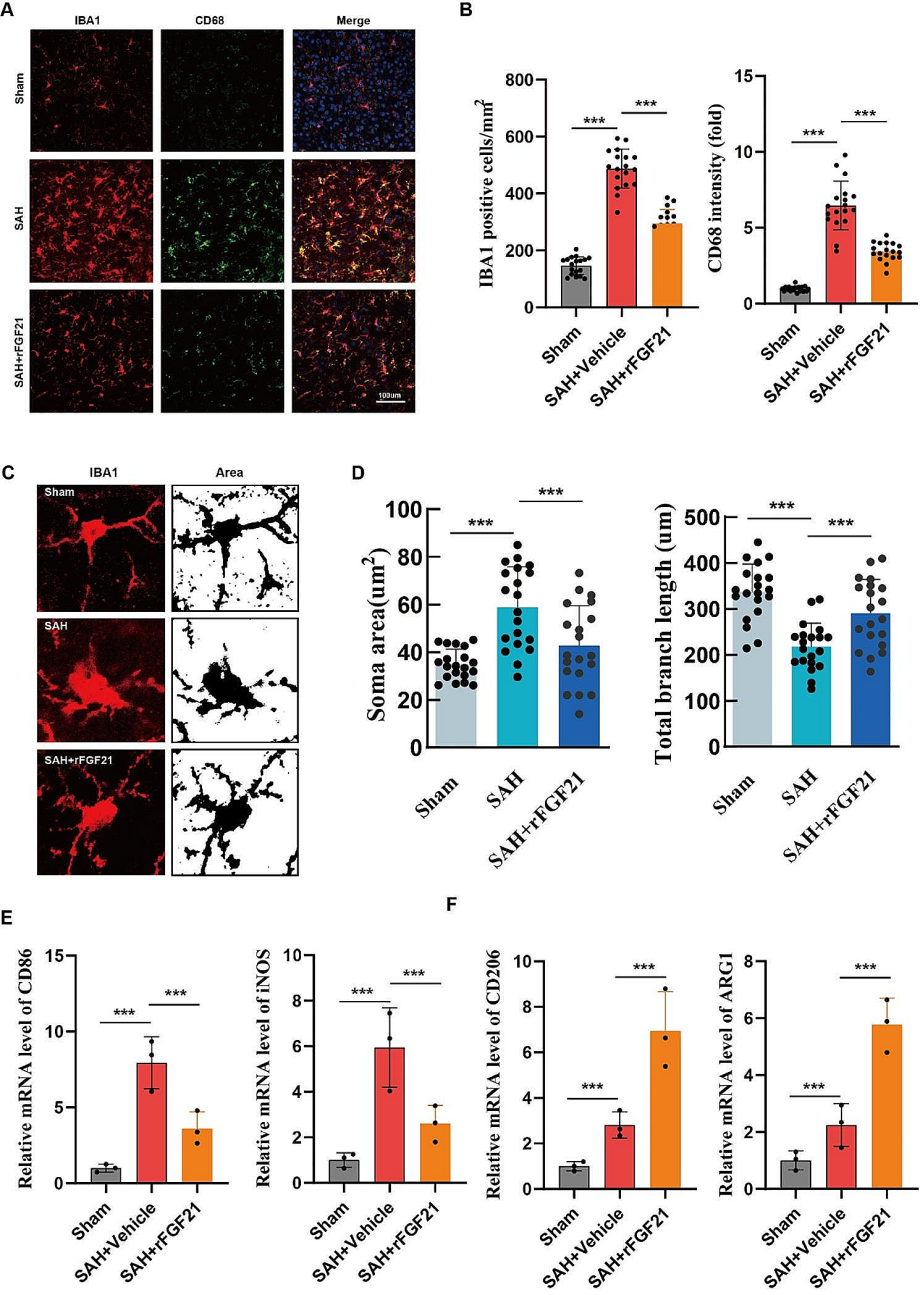
FGF21 inhibited microglial pro-inflammatory response after SAH. (**A-B**) Immunofluorescence staining for Iba1(red) with CD68 (green) revealing the activated microglia levels in each group (*n* = 18 from 6 mice per group). (**C-D**) Statistical analysis of total branch length (micrometers), and soma area (square micrometers) of Iba1-positive cells in each group (*n* = 18 from 6 mice per group). (**E-F**) qPCR analysis of M1 microglia-specific transcripts (CD86, iNOS) and of M2 microglia-specific transcripts (CD206, Arg1) in isolated microglia using flow cytometry. Data are presented as means ± SD. *p < 0.05, **p < 0.01, ***p < 0.001

Microglia, the predominant immune cells in CNS, play a crucial role in the regulation of neuroinflammation following SAH. We found that the density of CD68, a marker of hyperactivated microglia, noticeably increased after SAH, but was subsequently reversed with rFGF21 treatment (Fig. 3A-B). Additionally, we employed the ImageJ software to delineate and skeletonize microglial morphology through Iba1 staining. The SAH group exhibited a significant decrease in branch numbers and branch length, while also showing an increase in microglial soma area compared to the sham group, suggesting the hyperactivated state of microglia. However, rFGF21 treatment effectively rescued the SAH-induced morphological alterations (Fig. 3C-D). Considering that different microglial phenotypes are associated with either cytotoxic or neuroprotective effects, we further examined the impact of rFGF21 treatment on microglial phenotype switching. Microglia were isolated by flow cytometry and subjected to qPCR analysis. Notably, rFGF21 treatment resulted in a significant downregulation of M1 microglia-specific transcripts (CD86, iNOS) and an upregulation of M2 microglia-specific transcripts (CD206, Arg1) following SAH (Fig. 3E-F). Collectively, these findings indicate that rFGF21 treatment suppresses microglial proinflammatory activities subsequent to SAH.

### rFGF21 inhibits the activation of cGAS-STING pathway following SAH

**Fig. 4:**
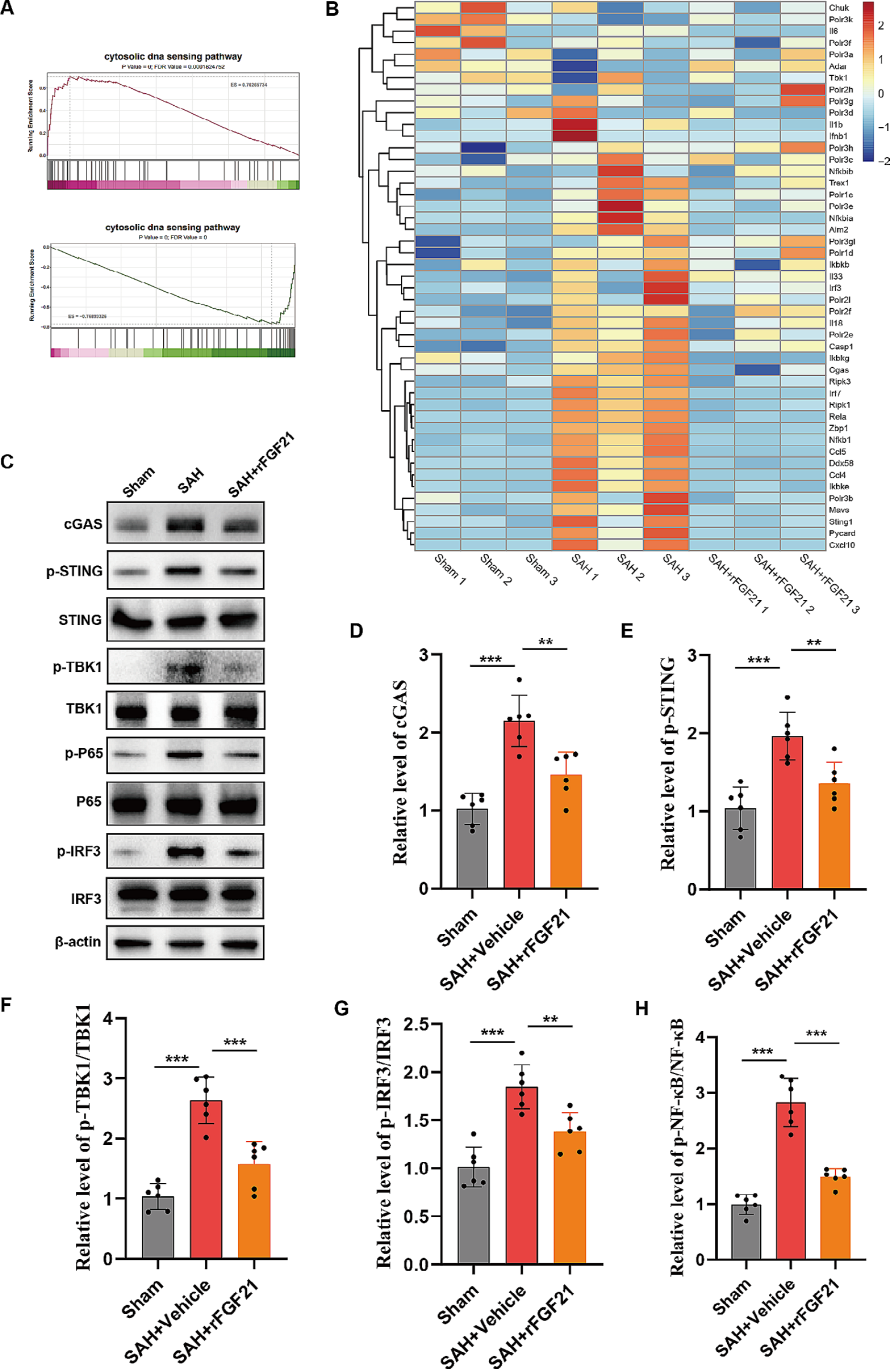
rFGF21 inhibits the activation of cGAS-STING pathway following SAH. (**A**) GSEA plot showing that the activation of cytosolic dna sensing pathway were activated in the brain from SAH mice, which were then alleviated following rFGF21 treatment. (**B**) Heatmap showing that differently expressed genes involved in cytosolic DNA sensor pathway. (**C-H**) Western blot analysis of the effects of rFGF21 treatment on the expression level of cGAS and the phosphorylation level of STING, TBK1, IRF3, and NF-κB following SAH operation in mice. (*n* = 6 per group). Data are presented as means ± SD. *p < 0.05, **p < 0.01, ***p < 0.001

Notably, transcriptomic analysis unveiled a substantial attenuation of various immune signature genes upon rFGF21 treatment, including those associated with type I IFN signaling and cytosolic DNA-sensing pathways (Fig. 4A-B). The cytosolic DNA sensor, cGAS, is known to recognize cytosolic dsDNA, thereby initiating type I interferon and NF-κB pathways. Western blot analysis revealed an upregulation of cGAS protein levels and STING phosphorylation levels following SAH (Fig. 4C-E). Consistent with the elevated expression of cGAS-STING, phosphorylated levels of TBK1, NF-κB, and IRF3 were significantly increased post-SAH (Fig. 4F-H). Importantly, FGF21 treatment significantly decreased the SAH-induced activation of cGAS-STING signaling (Fig. 4F-H). Collectively, rFGF21 inhibits cGAS-STING pathway mediated inflammatory response following SAH.

### rFGF21 attenuates cGAS-STING pathway mediated inflammatory response following SAH

**Fig. 5:**
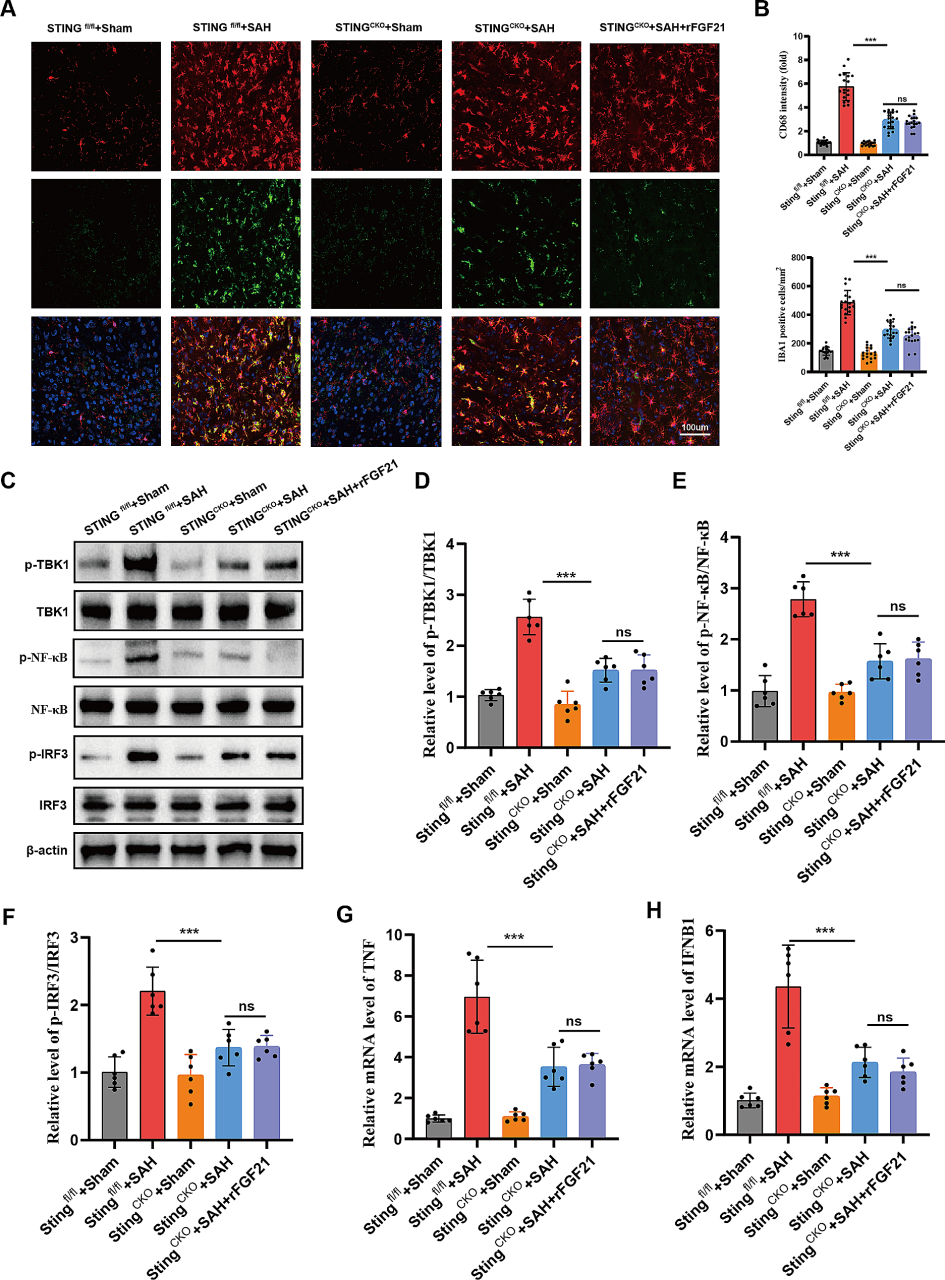
rFGF21 attenuates SAH-induced neuroinflammation through inhibition of cGAS-STING pathway. (**A-B**) Immunofluorescence staining for Iba1(red) with CD68 (green) in Sting^fl/fl^ and Sting ^CKO^ mice after SAH (*n* = 6 per group). (**C-F**) Western blot analysis of the phosphorylation level of TBK1, IRF3, and NF-κB in Sting^fl/fl^ and Sting ^CKO^ mice after SAH (*n* = 6 per group). (**G-H**) qPCR quantification of TNF and IFNB1 mRNA in Sting^fl/fl^ and Sting ^CKO^ mice after SAH (*n* = 6 per group). Data are presented as means ± SD. *p < 0.05, **p < 0.01, ***p < 0.001

In order to investigate the role of the cGAS-STING pathway in SAH-induced neuroinflammation and brain injury, we generated Sting^f/f^:CX3CR1creER^/+^ mice by crossing Sting^f/f^ mice with CX3CR1creER^/+^ mice. Through the administration of tamoxifen, we induced the conditional knockout (cKO) of Sting specifically in microglia. Subsequently, we subjected both Sting^fl/fl^ and Sting^CKO^ mice to SAH injury, and observed that compared to the Sting^fl/fl^ mice, the density of activated microglia significantly decreased in Sting^CKO^ mice after SAH (Fig. 5A-B). And Sting cKO led to a substantial downregulation of phosphorylated levels of TBK1, NF-κB, and IRF3 (Fig. 5C-F) and a remarkable reduction in the IFN-β and TNF mRNA levels in mice following SAH (Fig. 5G-H). These findings underscore the critical role of the cGAS-STING pathway in SAH-induced neuroinflammation. Additionally, we observed that rFGF21 treatment failed to further alleviate neuroinflammation in Sting^CKO^ mice (Fig. 5A-H), suggesting that rFGF21 attenuates SAH-induced neuroinflammation through inhibition of cGAS-STING pathway.

### rFGF21 attenuates SAH-induced brain injuries through inhibition of cGAS-STING pathway

**Fig. 6:**
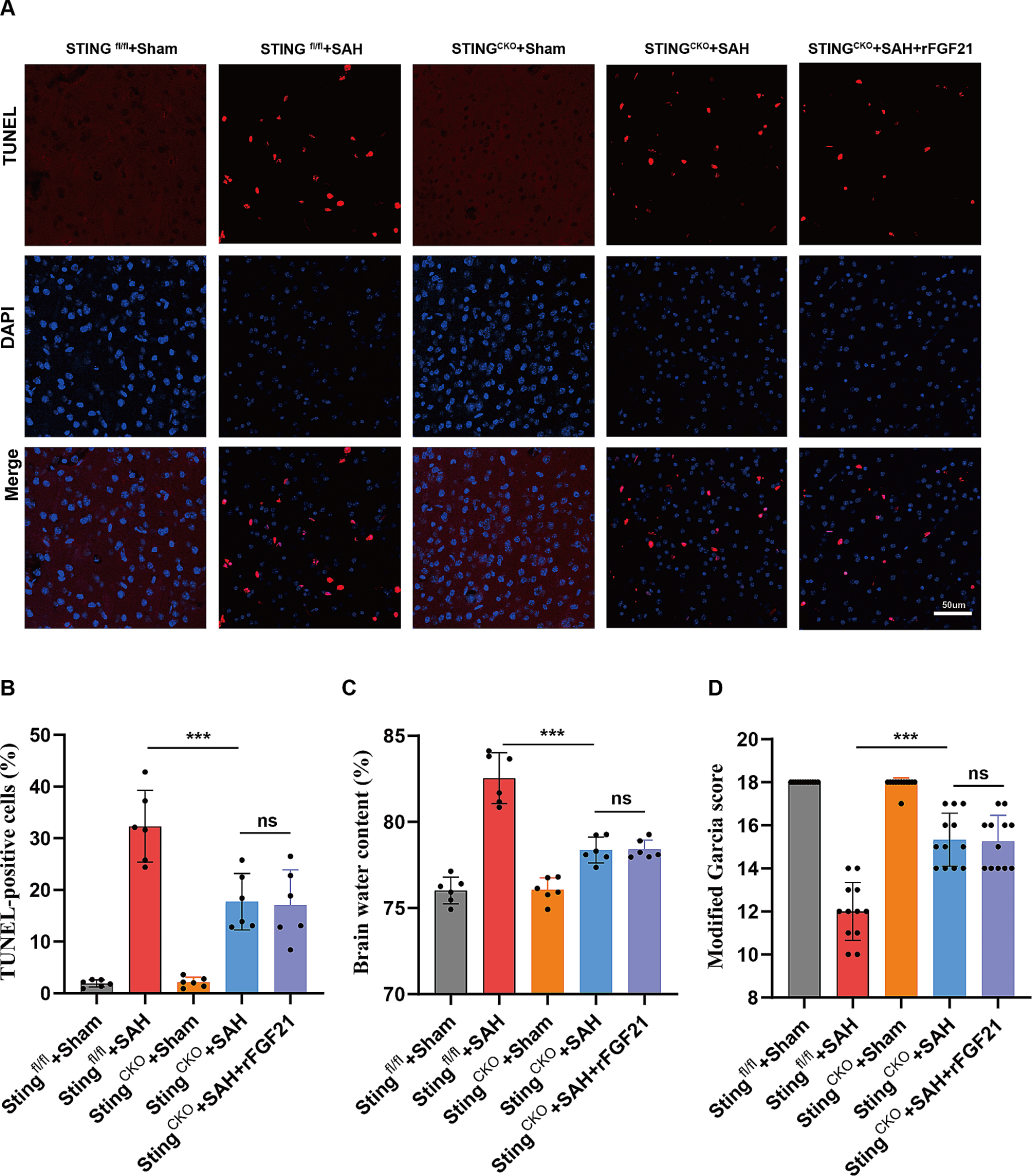
rFGF21 attenuates SAH-induced brain injuries through inhibition of cGAS-STING pathway. (**A-B**) Representative TUNEL staining images and quantification of TUNEL-positive cells in Sting^fl/fl^ and Sting ^CKO^ mice after SAH. (*n* = 6 per group). (**C**) Brain edema statistical analysis in Sting^fl/fl^ and Sting ^CKO^ mice after SAH. (**D**) Modified Garcia score of Sting^fl/fl^ and Sting ^CKO^ mice after SAH (*n* = 12 per group). Data are presented as means ± SD. *p < 0.05, **p < 0.01, ***p < 0.001

Tunel staining revealed a significant reduction in SAH-induced neuronal apoptosis upon microglial deletion of Sting (Fig. 6A-B). Additionally, brain water measurement demonstrated a mitigated brain edema in Sting^CKO^ mice as compared to Sting^fl/fl^ mice (Fig. 6C). Furthermore, Sting^CKO^ SAH mice exhibited superior performance in modified Garcia tests when compared to Sting^fl/fl^ SAH mice (Fig. 6D). However, the administration of rFGF21 failed to further alleviate brain injuries in Sting^CKO^ mice (Fig. 6A-D). Collectively, these findings suggest that rFGF21 attenuates SAH-induced cerebral injuries through inhibition of cGAS-STING pathway.

### FGF21 inhibited activation of cGAS-STING pathway by inducing mitophagy

**Fig. 7:**
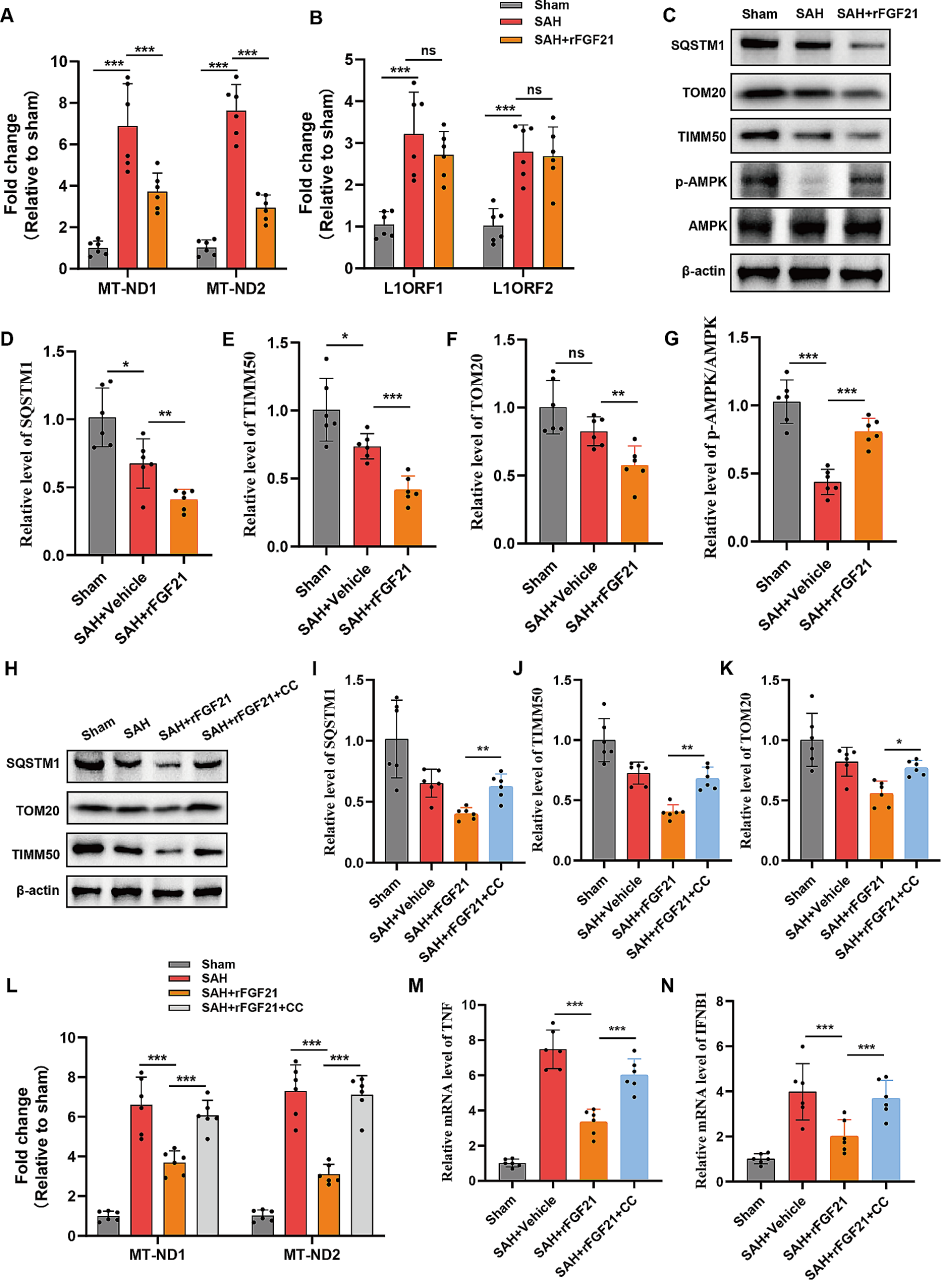
FGF21 inhibited activation of cGAS-STING pathway through inducing mitophagy and reducing cytosolic mtDNA. (**A**) Measurement of the cytosolic level of mtDNA (MT-ND1 and MT-ND2) through qPCR in isolated microglia from SAH mice with or without rFGF21 treatment (*n* = 6 per group). (**B**) Measurement of the cytosolic level of DNA corresponding to abundant nuclear LINE1 elements (L1ORF1 and L1ORF2) through qPCR in isolated microglia from SAH mice with or without rFGF21 treatment (*n* = 6 per group). (**C-G**) Western blot analysis of mitophagy related proteins in isolated microglia from SAH mice with or without rFGF21 treatment (*n* = 6 per group). (**H-K**) Inhibition of AMPK diminished the promotive effects of rFGF21 treatment on mitophagy (*n* = 6 per group). CC: compound C (inhibitor of AMPK pathway). (**L**) Inhibition of AMPK reversed the decreased cytosolic level of mtDNA induced by rFGF21 treatment (*n* = 6 per group). (**M-N**) Inhibition of AMPK diminished the suppression of rFGF21 treatment on TNF and IFNB1 mRNA expressions (*n* = 6 per group). Data are presented as means ± SD. *P < 0.05, **P < 0.01, ***P < 0.001

cGAS-STING pathway can be triggered by self-DNA that is released from damaged mitochondria and nuclei (35). We verified that there was a significant upregulation of cytoplasmic mtDNA in isolated microglia following SAH, which was subsequently mitigated by the administration of rFGF21(Fig. 7A). Moreover, we did not observe any substantial alterations in cytosolic DNA originating from the nucleus following rFGF21 treatment (Fig. 7B). These results suggested that FGF21 inhibited the activation of cGAS-STING pathway by preventing the accumulation of mtDNA in cytosolic. Impairment of mitophagy has been demonstrated to be an important reason for the accumulation of mtDNA in the cytoplasm. We hypothesized that FGF21 may inhibit the activation of cGAS-STING pathway by inducing mitophagy. As expected, the level of mitophagy in microglia isolated from SAH mouse brains treated with rFGF21 was elevated, as evidenced by the decrease in SQSTM1, TOMM20 and TIM50 levels (Fig. 7C-F). (AMP-activated protein kinase) AMPK is a well-established downstream effector of FGF21 [17, 37]. Treatment with rFGF21 resulted in an elevation in the phosphorylated form of AMPK (Fig. 7C and G). Furthermore, the inhibition of AMPK diminished FGF21-mediated promotion of mitophagy (Fig. 7H-K), reduction of cytoplasmic DNA (Fig. 7L) and suppression of IFN-β and TNF mRNA expressions (Fig. 7M-N) in isolated microglia. These findings strongly suggest that FGF21 enhances the process of mitophagy, leading to a decrease in the leakage of mtDNA, and concurrently inhibits cGAS-STING-dependent neuroinflammation.

## Discussion

The current study aimed to elucidate the therapeutic potential of FGF21 in the management of brain injury induced by SAH, along with its underlying molecular mechanisms. Mice treated with recombinant FGF21 displayed a notable reduction in neural apoptosis and brain edema post-SAH, accompanied by a significant improvement in neurological deficits. Transcriptomic analysis and subsequent experimental investigations revealed that recombinant FGF21 treatment substantially suppressed microglia-mediated neuroinflammation post-SAH. Remarkably, our study identified that the inflammatory response in SAH could be modulated through the cGAS-STING pathway, and treatment with recombinant FGF21 mitigated neuroinflammation and brain injuries in SAH mice by inhibiting cGAS-STING signaling. Additionally, we demonstrated that FGF21 impeded the activation of the cGAS-STING pathway by inducing mitophagy. In conclusion, our results suggest that FGF21 shows promising potential as a therapeutic agent for neuroinflammatory injury post-SAH, exerting its effects, at least partially, through the inhibition of the cGAS-STING pathway.

FGF21 is a widely recognized metabolic modulator that facilitates glucose and lipid metabolism. Strikingly, there has been a recent surge of multiple reports elucidating its neuroprotective advantages. Lin, for instance, unveiled how FGF21 fosters functional recuperation subsequent to ischemic brain injury via the activation of FGFR1/β-klotho/Akt signaling pathway [35] Furthermore, another investigation substantiated that recombinant FGF21 shields against blood-brain barrier permeability by upregulating Nrf2 in type 2 diabetes mice [36]. Nevertheless, the potential impact of FGF21 on SAH remains unexplored. Our findings unmasked that treatment with rFGF21 significantly mitigated apoptosis, cerebral edema, and facilitated neurological recovery following SAH. Consequently, our findings provide compelling evidences for FGF21 as a promising therapeutic agent for SAH.

Neuroinflammation plays a pivotal role in the pathogenesis of EBI subsequent to SAH [38]. Shortly after the occurrence of SAH, neuroinflammation promptly ensues, characterized by glial cell activation and the release of pro-inflammatory cytokines, matrix metalloproteinases, cytotoxic factors, and more [39]. Persistent neuroinflammation disrupts the integrity of BBB, leading to neuronal death and exacerbating the persistence of secondary brain injury [10]. In our study, utilizing RNA sequencing analysis, we unveiled that SAH-induced neuroinflammation was markedly attenuated following administration of rFGF21, underscoring its potent anti-inflammatory properties. Notably, our results are consistent with a prior investigation that highlighted FGF21 as a promising anti-inflammatory agent in ischemic stroke. Moreover, our subsequent investigations elucidated that the cGAS-STING pathway may orchestrate neuroinflammation, and treatment with rFGF21 effectively ameliorated neuroinflammation and mitigated brain damage in SAH mice by suppressing cGAS-STING signaling. Mounting evidence has illuminated the expanding role of the cGAS-STING pathway in immune responses against microbial infections, cellular senescence, antitumor immunity, inflammatory conditions, and autophagy. In our study, we have confirmed that the cGAS-STING pathway also contributes to the development of early brain injury in SAH. Significantly, the anti-inflammatory efficacy of rFGF21 treatment is, at least partially, dependent on the inhibition of the cGAS-STING pathway.

Investigating the mechanisms underlying the activation and regulation of cGAS-STING pathway, mtDNA plays a central role in activating the cGAS–STING signaling pathway, and disrupted mitochondrial homeostasis is a characteristic feature of SAH pathology. Notably, in SAH mice, microglial cells exhibited a significant accumulation of mtDNA in cytoplasm compared to Sham mice. Previous researches have indicated alternative mechanisms through which mtDNA can be leakaged into the cytoplasm, such as TFAM deficiency [40] or opening of mitochondrial permeability transition pore [41, 42]. A recent investigation demonstrated that a deficiency in mitophagy promoted the release of mtDNA and cGAS-STING pathway activation in murine cardiac tissue, ultimately resulting in a potent inflammatory phenotype [43]. And previous studies have documented impaired mitophagy in microglia following stroke [44]. Importantly, our findings demonstrate that FGF21 reduces the presence of cytoplasmic DNA in the context of SAH by inducing mitophagy. We propose that the deficiency of mitophagy in microglia following SAH leads to an increased release of DNA into the cytosol, thereby promoting the activation of cGAS–STING and subsequent aberrant neuroinflammation. AMPK plays a crucial role in regulating energy expenditure and may also be implicated in the process of mitophagy [45, 46]. Furthermore, it has been reported that FGF21 modulates mitochondrial quality control through the AMPK pathway. In our study, we observed that the inhibition of AMPK abrogated the promoting effects of FGF21 on mitophagy and its inhibitory effects on cGAS–STING, suggesting that FGF21 functions in an AMPK-dependent manner.

Numerous constraints warrant acknowledgment. Primarily, our investigation singularly concentrated on delineating the protective efficacy of rFGF21 against initial-phase cerebral injury subsequent to SAH, while its impact during the latter phase remains ambiguous. Secondly, our experimental paradigm utilized juvenile and robust animal subjects devoid of exposure to alternative pharmaceutical agents. Nevertheless, SAH patients typically manifest with profound comorbidities such as diabetes and cardiovascular ailments, frequently necessitating pre-administration of diverse medications. Consequently, forthcoming inquiries should duly consider the potential influence of these pharmacological interventions.

In summary, our research furnishes compelling substantiation that FGF21 confers a neuroprotective influence by ameliorating neuroinflammation and mitigating cerebral injury subsequent to SAH, predominantly via the inhibition of the cGAS-STING pathway.

## Data Availability

The datasets used and/or analyzed during the current study are available from the corresponding author on reasonable request.

## Abbreviations

SAH: Subarachnoid hemorrhage
FGF21: Fibroblast growth factor 21
IFN-I: type I IFN
mtDNA: mitochondrial DNA
EBI: early brain injury
dsDNA: double-stranded DNA
AMPK: AMP-activated protein kinase
cGAS: cyclic GMP-AMP synthase
